# Autoimmunity to synovial extracellular matrix proteins in patients with postinfectious Lyme arthritis

**DOI:** 10.1172/JCI161170

**Published:** 2023-09-01

**Authors:** Korawit Kanjana, Klemen Strle, Robert B. Lochhead, Annalisa Pianta, Laura M. Mateyka, Qi Wang, Sheila L. Arvikar, David E. Kling, Cameron A. Deangelo, Lucy Curham, Alan G. Barbour, Catherine E. Costello, James J. Moon, Allen C. Steere

**Affiliations:** 1 Center for Immunology and Inflammatory Diseases, Division of Rheumatology, Allergy, and Immunology, Massachusetts General Hospital, Harvard Medical School, Boston, Massachusetts, USA.; 2 Center for Biomedical Mass Spectrometry, Boston University Chobanian & Avedisian School of Medicine, Boston, Massachusetts, USA.; 3 Department of Microbiology and Molecular Genetics, University of California, Irvine, Irvine, California, USA.

**Keywords:** Autoimmunity, Infectious disease, Adaptive immunity, Extracellular matrix, T cells

## Abstract

**BACKGROUND:**

Autoimmune diseases often have strong genetic associations with specific HLA-DR alleles. The synovial lesion in chronic inflammatory forms of arthritis shows marked upregulation of HLA-DR molecules, including in postinfectious Lyme arthritis (LA). However, the identity of HLA-DR–presented peptides, and therefore the reasons for these associations, has frequently remained elusive.

**METHODS:**

Using immunopeptidomics to detect HLA-DR–presented peptides from synovial tissue, we identified T cell epitopes from 3 extracellular matrix (ECM) proteins in patients with postinfectious LA, identified potential *Borreliella* *burgdorferi*–mimic (*Bb*-mimic) epitopes, and characterized T and B cell responses to these peptides or proteins.

**RESULTS:**

Of 24 postinfectious LA patients, 58% had CD4^+^ T cell responses to at least 1 epitope of 3 ECM proteins, fibronectin-1, laminin B2, and/or collagen Vα1, and 17% of 52 such patients had antibody responses to at least 1 of these proteins. Patients with autoreactive T cell responses had significantly increased frequencies of HLA-DRB1*04 or -DRB1*1501 alleles and more prolonged arthritis. When tetramer reagents were loaded with ECM or corresponding *Bb*-mimic peptides, binding was only with the autoreactive T cells. A high percentage of ECM-autoreactive CD4^+^ T cells in synovial fluid were T-bet–expressing Th1 cells, a small percentage were RoRγt-expressing Th17 cells, and a minimal percentage were FoxP3-expressing Tregs.

**CONCLUSION:**

Autoreactive, proinflammatory CD4^+^ T cells and autoantibodies develop to ECM proteins in a subgroup of postinfectious LA patients who have specific HLA-DR alleles. Rather than the traditional molecular mimicry model, we propose that epitope spreading provides the best explanation for this example of infection-induced autoimmunity.

**FUNDING:**

Supported by National Institute of Allergy and Infectious Diseases R01-AI101175, R01-AI144365, and F32-AI125764; National Institute of Arthritis and Musculoskeletal and Skin Diseases K01-AR062098 and T32-AR007258; NIH grants P41-GM104603, R24-GM134210, S10-RR020946, S10-OD010724, S10-OD021651, and S10-OD021728; and the G. Harold and Leila Y. Mathers Foundation, the Eshe Fund, and the Lyme Disease and Arthritis Research Fund at Massachusetts General Hospital.

## Graphical abstract

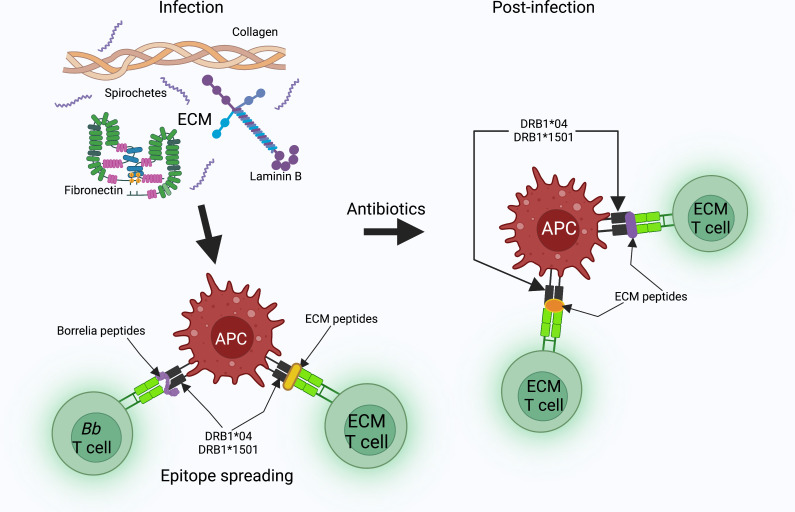

## Introduction

Lyme arthritis (LA), which is caused by infection with the tick-borne spirochete *Borreliella* (*Borrelia*) *burgdorferi* (*Bb*), is typically manifested by swelling and pain in a few large joints, especially one or both knees (1). The arthritis can usually be treated successfully with oral and, if necessary, with i.v. antibiotic therapy, called antibiotic-responsive LA (2–4). However, in some patients, the arthritis changes after antibiotic therapy, and a persistent proliferative synovitis develops. PCR and culture results for *Bb* have been uniformly negative in synovial tissue from such patients (5), which we have termed postinfectious LA (also called antibiotic-refractory or postantibiotic LA).

The basic pathologic feature of postinfectious LA is the development of an excessive, dysregulated proinflammatory immune response during the infection characterized by high IFN-γ levels and inadequate levels of the antiinflammatory cytokine IL-10, which persists in the postinfectious period (6). The consequences of this excessive proinflammatory response in Lyme synovia include vascular damage, autoimmune and cytotoxic responses, and massive fibroblast proliferation and fibrosis. This lesion is similar to that seen in other forms of chronic inflammatory arthritis, including rheumatoid arthritis (RA).

Fibroblast-like synoviocytes (FLSs) are the predominant cell type in the synovial lesions of patients with chronic inflammatory arthritis, including in those with postinfectious LA or RA (6). In RA, multiple types of FLS have been identified, including fibroblasts that secrete and degrade extracellular matrix (ECM) proteins, and “immune-interacting” FLSs (iFLSs) that activate and modulate immune responses to pathologic stimuli (7). In postinfectious LA patients, FLSs secrete a range of arthritogenic mediators, including Th1-promoting immune reactants, and they upregulate marked cell surface expression of antigen-presenting molecules, including HLA-DR and costimulatory molecules (8).

Autoimmune diseases often have strong genetic associations with specific HLA-DR alleles (9, 10). Based on findings in historic LA patients, sometimes seen prior to the use of antibiotics for treatment of the disease, we previously reported increased frequencies of the HLA-DR4 specificity and secondarily of the DR2 specificity (now called the DRB1*1501) in patients with the most prolonged courses (11). In a later study performed with molecular techniques, HLA-DR molecules that bound a specific epitope of *Bb* outer-surface protein A (OspA^163–175^), particularly DRB1*04 molecules, were increased in frequency in patients with antibiotic-refractory LA (called here postinfectious LA) (12). Among patients with 1 OspA^163–175^ epitope–binding HLA-DR molecule, the odds ratio of having antibiotic-refractory LA was 4.4, and among patients with 2 such HLA-DR molecules, it was 11.3 times that in patients with antibiotic-responsive LA. However, in most autoimmune diseases, the peptides presented by disease-associated HLA-DR molecules, and therefore the reasons for these associations, have frequently remained elusive.

Recent discovery-based techniques have offered new approaches to this problem. We developed a novel, discovery-based immunopeptidomics approach in which HLA-DR–presented peptides are identified directly from patients’ synovial tissue by nanoscale liquid chromatography–tandem mass spectrometry (nano-LC-MS/MS), followed by determination of the antigenicity of the phenotype-specific peptides and their proteins of origin using patient samples (13, 14). With this approach, we previously reported the identification of 4 autoantigens in Lyme synovia: 3 related to the vasculature, endothelial cell growth factor (ECGF), annexin A2, and apolipoprotein B-100 (apoB-100), and 1 ECM proteinase, matrix metalloproteinase 10 (MMP-10). These proteins are each targets of T and B cell responses that correlate with synovial pathology in subgroups of LA patients (15–19).

Here, we identified HLA-DR–presented peptides (T cell epitopes) of 3 ECM proteins presumably presented primarily by FLSs, determined possible *Bb*-mimic epitopes, characterized T and B cell immune responses to these peptides and proteins, and correlated T cell responses to ECM peptides with HLA-DR type and disease outcome.

## Results

### Initial identification of immunogenic HLA-DR–presented self-peptides in synovial tissue.

Previously, we identified about 100 to 800 HLA-DR–presented self-peptides from the synovial tissue of each of 8 patients with postinfectious LA using nano-LC-MS/MS. The complete list of peptides identified has been published previously (14). To determine antigenicity, each peptide was synthesized and tested for reactivity with the matching patient’s PBMCs using IFN-γ ELISpot assays. In our previous report, these determinations had been completed in the first 5 LA patients (14). For the current study, additional testing was done in the remaining 3 patients (LA6, LA7, and LA8), using newly available, more sensitive mass spectrometers and peptide-identification search algorithms.

**Figure 1:**
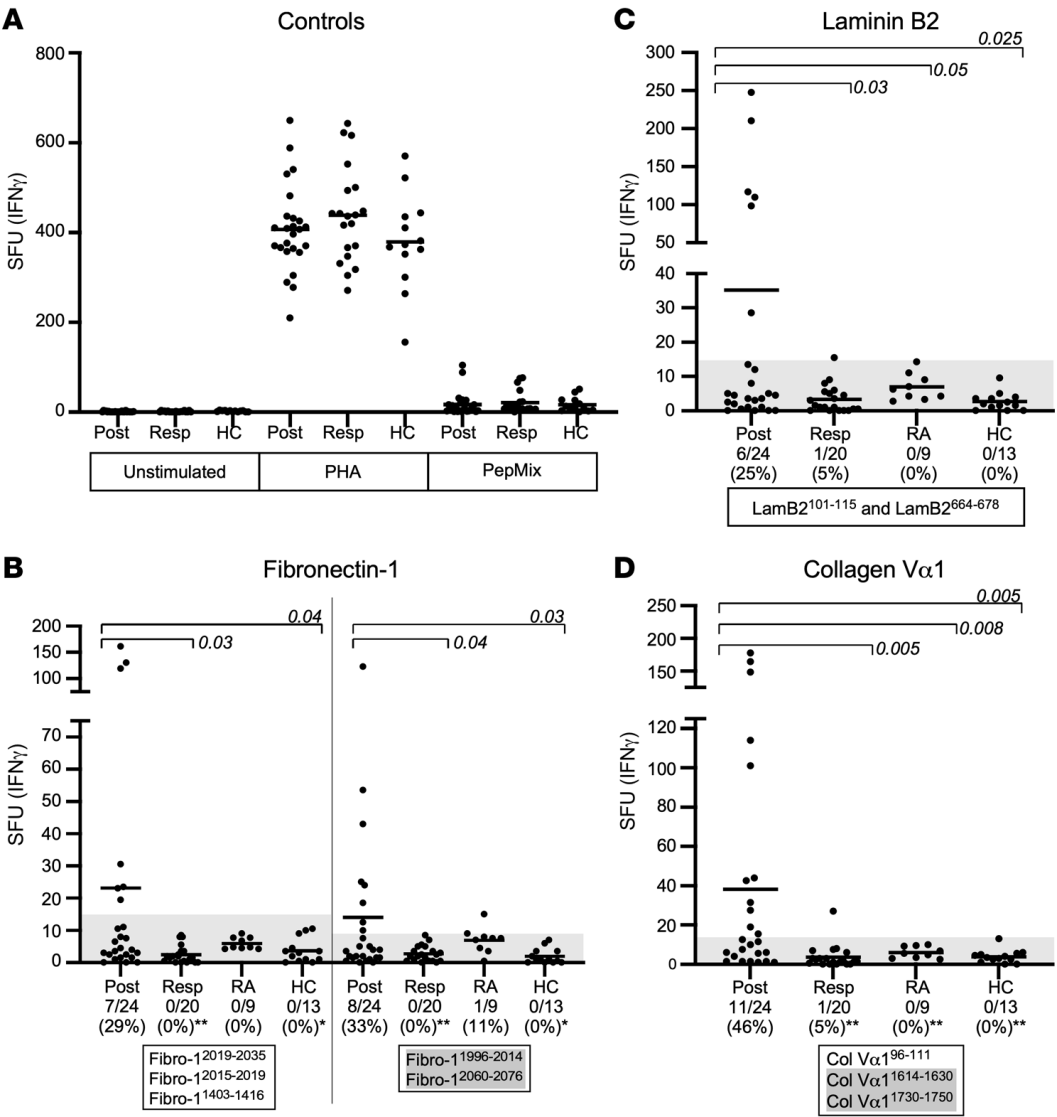
Autoreactive T cell responses to peptides from synovial ECM proteins stratified by patient groups. T cell reactivity with 10 ECM-derived peptides from fibronectin-1 (**B**), laminin B2 (**C**), or collagen Vα1 (**D**) was determined in patients with postinfectious LA (Post, *n* = 24) or antibiotic-responsive LA (Resp, *n* = 20), in patients with rheumatoid arthritis (RA, *n* = 9), and in healthy control subjects (HC, *n*-13), using IFN-γ ELISpot assays. As controls, PBMCs were unstimulated or stimulated with PHA or with PepMix (**A**). The 6 peptides identified from synovial tissue are shown with a white background, whereas the 4 epitopes identified with the IEDB are shown with a shaded background. A positive response was defined as a value more than 3 SD above the mean value for HC (the gray shaded region). Horizontal lines represent the mean values for each group. The number of patients with positive responses in each group was compared between patients with postinfectious LA and each of the other 3 groups. The distribution of values between groups was compared using an unpaired *t* test with Welch’s correction, and these *P* values, which are 2 tailed, are shown above the data points. The identity of groups was compared by Fisher’s exact test, and these *P* values, which are 2 tailed, are shown with asterisks below the data points. *P ≤ 0.05, **P ≤ 0.01. SFU, spot-forming units per million cells.

To conserve cells for subsequent experiments, HLA-DR–presented self-peptides identified in synovial tissue were first tested in pools containing 3–5 peptides, and if enough cells were still available, each peptide in reactive pools was then tested individually. In patient LA6, a total of 95 peptides were identified, and 3 pools containing a total of 9 peptides showed reactivity in IFN-γ ELISpot assays. When the 9 peptides were tested individually, 4 gave positive results (Table 1). In patient LA7, 285 HLA-DR–presented peptides were identified, and 6 pools containing 23 peptides showed reactivity. When tested individually, 6 of the 23 peptides gave positive results (Figure 1). In patient LA8, 732 HLA-DR–presented peptides were identified, and 5 pools containing 20 peptides were immunoreactive, but not enough cells remained to test all peptides individually. Taken together, 1,112 HLA-DR–presented peptides derived from 296 self-proteins were identified, and 30 candidate peptides (0.03%) were potentially immunogenic. Testing of all other peptides gave negative results.

Because of the large number of PBMCs required to test T cell reactivity of 30 candidate peptides in large numbers of patients as well as the need to conserve cells for other subsequent analyses, we selected only certain peptides of interest for detailed analysis. Because of the central importance of FLSs in chronic inflammatory arthritis, we chose to study herein HLA-DR–presented peptides from the 3 ECM proteins, fibronectin-1, laminin B2, and collagen Vα1, presumably derived from FLSs, which were identified directly from the synovial tissue of patient LA7 or patient LA8. The mass spectra for each of these peptides are shown in Supplemental Figure 1 (supplemental material available online with this article; https://doi.org/10.1172/JCI161170DS1). Although we did not isolate synovial fibroblasts from synovectomy samples before capturing HLA-DR–presented peptides, we have previously shown by histology that FLSs are by far the predominant cell type in the synovial lesions of postinfectious LA patients, and as many as half of these cells exhibit upregulation of HLA-DR molecules (8). Although dendritic cells and macrophages (professional antigen-presenting cells [APCs]) may present peptides derived from ECM proteins, many such peptides were likely presented by HLA-DR molecules on synovial fibroblasts (non-professional APCs).

The 3 patients from whom synovial tissue was obtained (LA6, LA7, and LA8) were young male teenagers who had high antibody responses to *Bb* with expansion of the response to many spirochetal proteins (Table 2). Because we usually follow an algorithm for the treatment of LA, their therapies were quite similar, including about 2 months of oral doxycycline, followed by 1 month of i.v. ceftriaxone, and then by 5 months of methotrexate. Because of incomplete responses and patient preference, they then underwent arthroscopic synovectomies. Patient LA6 had complete remission after synovectomy, whereas patients LA7 and LA8 continued to have arthritis in the affected knee for 18 or 10 months, respectively, after the procedure. Patients LA7 and LA8 had HLA-DR alleles associated with persistent LA after antibiotic therapy (11, 12), and they both had immunogenic HLA-DR–presented peptides derived from ECM proteins in synovial tissue. In contrast, patient LA6 did not have such alleles, and ECM peptides were not identified in his synovial tissue. Although LA7 and LA8 required longer to achieve remission, all 3 patients subsequently remained well.

Using epitope prediction tools from the Immune Epitope Database (IEDB), the 5 HLA-DR–presented peptides from ECM peptides identified in patients’ synovia were first analyzed for their predicted binding to HLA-DR molecules, and then other epitopes of the 3 ECM proteins were identified that were predicted to bind multiple, common HLA-DR molecules (Table 3). Altogether, a total of 10 T cell epitopes of the 3 ECM proteins were synthesized and tested. Six of the 10 epitopes were HLA-DR–presented peptides (T cell epitopes) identified directly from synovial tissue (2 of these peptides, fibronectin-1^2019–2035^ and fibronectin-1^2015–2029^, originated from the same peptide but had 2 predicted binding patterns). The remaining 4 peptides were predicted epitopes from these same ECM proteins identified from the IEDB database.

### Autoreactive T cell responses to synovial ECM peptides.

As the next step, T cell responses to the 10 synovial ECM peptides were assessed in larger numbers of LA patients and in individuals in comparison groups. Using IFN-γ ELISpot assays, T cell reactivity with the 10 peptides was determined from PBMCs of 24 patients with postinfectious LA, 20 patients with antibiotic-responsive LA, and 9 patients with RA, and from 13 healthy control subjects (Figure 1). To conserve cells for subsequent experiments, peptide epitopes from fibronectin-1, laminin B2, and collagen Vα1 were tested in pools containing 2 or 3 epitopes from the same protein.

Phytohemagglutinin (PHA), a T cell mitogen, induced strongly positive responses in cells from all patients and healthy control subjects; T cells stimulated with PepMix, a positive control containing antigens from commonly encountered infectious agents, such as influenza, induced low-level reactivity in cells from a few patients and control subjects. The negative control, which was unstimulated cells from patients and control subjects, showed no background reactivity (Figure 1A). Among LA patients, positive responses to T cell epitopes of the ECM proteins, which were defined as more than 3 SD above the mean value in 13 healthy control subjects, were found almost exclusively in patients with postinfectious LA. Among 24 postinfectious LA patients, 29% and 33%, respectively, had responses to one or both pools of fibronectin peptides, 25% had reactivity with the laminin B2 peptides, and 46% had responses to the pool of collagen Vα1 peptides (Figure 1, B–D). In contrast, only 1 patient with antibiotic-responsive LA had borderline positive responses to laminin B2 and collagen Vα1 peptides. Of 9 patients with RA, all of whom had an HLA-DRB1*0401 or 1501 allele, only 1 had borderline reactivity with fibronectin-1 peptides but no response to either the laminin B2 or collagen Vα1 peptides. Altogether, 14 of 24 patients (58%) with postinfectious LA had T cell responses to at least 1 ECM peptide compared with borderline positive responses in only 1 of 20 patients (5%) with antibiotic-responsive LA (*P* = 0.0001) and in only 1 of 9 RA patients (*P* = 0.02).

### Clinical correlations.

The median age of patients with postinfectious LA was significantly younger than that of patients with antibiotic-responsive LA (*P* < 0.001), though the age ranges (from young teenagers to older adults) were similar in the 2 groups (Table 4). Because most patients with LA have been White, the patient population tested here consisted of White men and women. As one would expect, patients with postinfectious LA, who typically received two 1-month courses of oral antibiotics and a 1-month course of i.v. antibiotics, had a significantly longer duration of treatment than patients with antibiotic-responsive LA, who usually responded to only 1 course of oral antibiotics (*P* < 0.0001). Similarly, the total duration of arthritis in the postinfectious group was significantly longer than that in the antibiotic-responsive group (*P* < 0.0001).

When patients with postinfectious LA were stratified according to whether they had T cell responses to ECM peptides, the subgroup with these responses had a significantly longer duration of postinfectious arthritis compared with that in patients who lacked such immune responses (*P* = 0.05) (Table 4). In addition, the subgroup with ECM-peptide reactivity tended to have a longer duration of arthritis prior to antibiotic treatment (*P* = 0.06) and a longer total duration of arthritis than those in patients who lacked these immune responses (*P* = 0.1).

Importantly, T cell responses to ECM peptides correlated with HLA-DR alleles that were first associated years ago with LA of prolonged duration (11). Of the 24 current patients with postinfectious LA, 13 (54%) with responses to ECM peptides had HLA-DRB1*04 alleles (0401, 0402, 0403, 0404, 0407, 0408) and/or the DRB1*1501 allele (Table 5). Moreover, postinfectious LA patients with such responses had a higher frequency of DRB1*04 alleles (*P* = 0.05) and a trend in that direction for the DRB1*1501 allele compared with postinfectious patients who lacked these immune responses (*P* = 0.1). In comparison, only 1 of 20 antibiotic-responsive patients (5%) had borderline positive responses to ECM proteins (*P* = 0.0008). That patient, a 59-year-old man, also had the DRB1*0402 and 1501 alleles. Within 1 week after onset of arthritis, he was treated with a 1-month course of doxycycline, and his arthritis resolved, suggesting that early antibiotic treatment was a factor in his antibiotic-responsive course.

### IgG autoantibody responses to ECM proteins.

**Figure 2:**
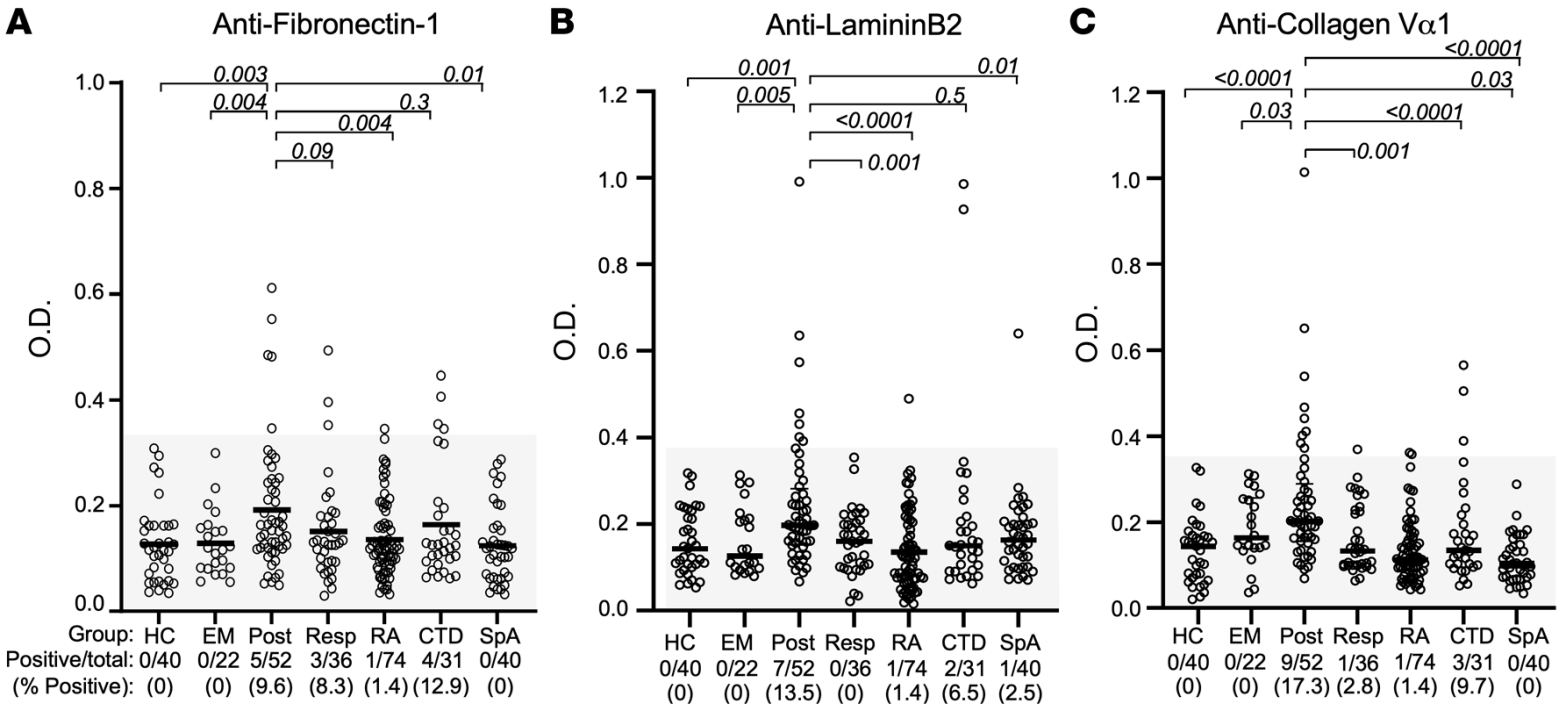
IgG autoantibody responses to synovial ECM proteins in case and control subjects. Serum autoantibodies against fibronectin-1 (**A**), laminin B2 (**B**), and collagen Vα1 (**C**) were measured in patients with various manifestations of Lyme disease, erythema migrans (EM, *n* = 22), antibiotic-responsive LA (Resp, *n* = 36), or postinfectious LA (Post, *n* = 52), as determined by ELISA. For comparison, antibody responses were measured in patients with other rheumatic diseases: RA (*n* = 74), spondyloarthropathy (SpA, *n* = 40), or other connective tissue diseases (CTD, *n* = 31), including systemic lupus, mixed CTD, scleroderma, or Sjögren’s syndrome; and in healthy control subjects (HC). A positive response was defined as a value more than 3 SD above the mean OD value for healthy controls (area above the gray shaded region). Horizontal lines represent the mean values. *P* values were determined by unpaired *t* test with Welch’s correction. *P* values are 2-tailed; values less than or equal to 0.05 were considered statistically significant.

Using recombinant preparations of the 3 ECM proteins, IgG antibody responses to each protein were determined by ELISA in serum samples from 52 patients with postinfectious LA, 36 with antibiotic-responsive LA, and 22 with erythema migrans (EM), the initial skin lesion of Lyme disease (Figure 2). For comparison, sera were tested from 74 patients with RA, 40 with spondyloarthropathies (SpA), and 31 with other connective tissue diseases. A positive response was defined as more than 3 SD above the mean value in 40 healthy control subjects.

Of the 52 patients with postinfectious LA, 5 (10%) had anti–fibronectin-1 antibodies, 7 (14%) had anti–laminin B2 antibodies, and 9 (17%) had anti–collagen Vα1 antibodies (Figure 2). Moreover, postinfectious LA patients had significantly higher mean values or a trend in that direction compared with the values in the other groups. In contrast, only a few patients with antibiotic-responsive LA and no patients with EM had positive responses. Similarly, a few patients with RA or SpA had antibody responses above the cutoff value for fibronectin-1, laminin B2, or collagen Vα1, and several patients with other connective tissue diseases had positive values for at least 1 of these proteins, including 2 with polymyalgia who had high anti–laminin B2 values. Altogether, 9 of the 52 patients (17%) with postinfectious LA had responses to at least 1 of the 3 proteins compared with 4 of the 36 patients (11%) with antibiotic-responsive LA (*P* = NS) and compared with none of the 22 EM patients (*P* = 0.05). Of the patients with postinfectious LA from whom samples were available to do both T cell and antibody testing, 3 of 5 patients (60%) had both responses to fibronectin-1, 6 of 8 patients (75%) had both responses to collagen Vα1, and 2 of 6 patients (33%) had both responses to laminin B2. Since only 1 patient with antibiotic-responsive LA had low-level T cell reactivity with ECM proteins, this type of analysis could not be done with the responsive group.

### T cell responses to synovial ECM peptides and Bb-mimic peptides.

We next explored whether molecular mimicry between microbial and host T cell epitopes may be a mechanism linking infection and autoimmune responses (20). Using BLAST analysis, we searched for sequence alignment between the 3 synovial ECM peptides and *Bb-*mimic peptides. Candidate *Bb*-mimic peptides were then analyzed using the IEDB database for HLA-DR binding predictions and for physiochemical properties of mismatched amino acids in these sequences, such as charge, pH, and hydrophobicity, that might favor or negate peptide binding.

From this analysis, we identified 10 candidate ECM peptide–*Bb* mimic pairs (Supplemental Table 1). The most likely mimic pair (no. 9 in Supplemental Table 1) was a collagen Vα1^1730–1750^ epitope and an epitope of *Bb* protein, BBQ62^71–85^, which was annotated on linear plasmid 56 (lp56). However, in this study, this *Bb* peptide sequence was also found on lp28-2, and this previously non-annotated protein, which we called a BBQ62-like protein, was then annotated (GenBank accession number ONO23120). Although the BBQ62 and the BBQ62-like proteins have only 78% sequence identity, the *Bb*-mimic peptide from both proteins shares 7 amino acids with the collagen Vα1^1730–1750^ peptide, including all 5 amino acids responsible for peptide binding to the HLA-DR molecule. Three other possible ECM–*Bb* mimic pairs, fibronectin^2015–2029^ (pair 2), fibronectin^1403–1416^ (pair 4), and laminin B2^664–678^ (pair 7), shared 6 amino acids with a corresponding *Bb* protein, though they were predicted to be bound by fewer HLA-DR molecules. The remaining 6 ECM–*Bb* mimic pairs seemed unlikely to serve as molecular mimics.

**Figure 3:**
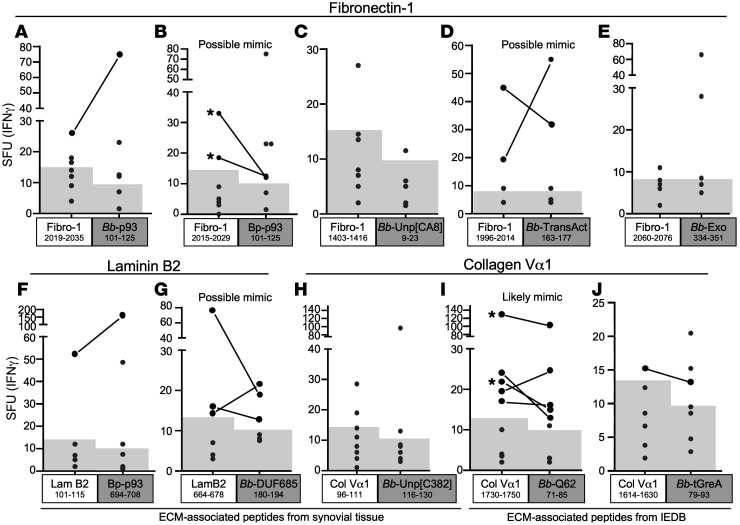
T cell responses to individual peptides of synovial ECM proteins and possible *Bb*-mimic peptides. PBMCs from postinfectious LA patients who had positive responses to any ECM-peptide pool were stimulated with individual ECM-derived peptides or their corresponding *Bb*-mimic peptides. T cell responses to the fibronectin-1 peptides (**A**–**E**), laminin B2 peptides (**F** and **G**), and collagen Vα1 peptides (**H**–**J**) and each corresponding *Bb*-mimic peptide were measured by IFN-γ ELISpot assay. In 1 ECM and borrelial peptide pair (**I**), the matches were close enough to be likely T cell epitope mimics; in 3 pairs, the matches were considered as possible mimics (**B**, **D**, and **G**); and in the remaining 6 pairs, epitope mimicry seemed unlikely. The location of the peptide in each protein is shown with the superscript numbers. A positive response was defined as a value more than 3 SD above the mean value for healthy controls (area above the gray shaded region). Paired-sample analysis was done using Wilcoxon’s rank-sum test. The asterisks in **B** and **I** identify patients with the HLA-DRB1*1501 allele who had responses to both ECM and *Bb*-mimic epitopes and were therefore selected for further studies using tetramer reagents, shown in Figures 4 and 5.

To compare reactivity among likely, possible, or unlikely ECM–*Bb* mimic pairs, we synthesized and tested peptides individually from all 10 pairs for reactivity with PBMCs from the 10 patients who had positive responses to peptide pools of fibronectin-1, laminin B2, or collagen Vα1 peptides, as determined with INF-γ ELISpot assays (shown in Figure 1). Consistent with IEDB predictions, 5 of 8 patients had reactivity with the most likely ECM–*Bb* mimic pair, collagen Vα1^1730–1750^ and BBQ62^71–85^ (Figure 3I). In addition, 2 or 3 patients each had responses to the 3 possible ECM–*Bb* mimic pairs. These included fibronectin^2015–2029^ and *Bb* p93^101–125^ (Figure 3B), fibronectin^1996–2014^ and *Bb* transcriptional activator protein^163–177^ (Figure 3D), and laminin B2^664–678^ and *Bb* DUF685 protein^80–194^ (Figure 3G). In comparison, only 2 patients’ cells had responses to 1 ECM-*Bb* pair (Figure 3, A and F), and no patients’ cells had reactivity with the other 4 ECM-*Bb* pairs (Figure 3, C, E, H, and J). Altogether, 8 of the 10 patients had T cell reactivity with 1 or more of the ECM–*Bb* mimic pairs.

An IgG antibody response to the 93 kDa *Bb* protein (also called P83/100), which includes a sequence that is a mimic of the fibronectin^2015–2029^ peptide, is found in the majority of LA patients (21), and reactivity with this *Bb* protein was noted on Western blots of all 24 current study patients with postinfectious LA. In contrast, in a previous study, none of 39 LA patients tested had IgG antibody responses to the lp56-encoded BBQ62 protein, which includes a mimic of the collagen Vα1^1730–1750^ peptide (22). For this study, serum samples from the 24 postinfectious LA patients were tested for IgG reactivity by ELISA with a recombinant lp28-2–encoded BBQ62-like protein. Of the 24 patients, only 1 had a response that was minimally higher than 3 SD above the mean value in 12 healthy control subjects (data not shown), suggesting that this collagen-BBQ62 example of T cell epitope mimicry is not immunologically relevant.

### HLA-DR tetramer epitope-specific CD4^+^ T cell identification from synovial fluid mononuclear cells.

To examine the frequencies of ECM-autoreactive CD4^+^ T cells and to further explore their potential for cross-reactivity between ECM and *Bb*-mimic peptides, we analyzed synovial fluid mononuclear cells (SFMCs), rather than PBMCs, from 4 patients who had the HLA-DRB1*1501 allele using tetramer reagents. Although SFMCs are less often available, they are presumably more representative of events at the site of infection. When their PBMCs had been tested by ELISpot assay, 2 of the 4 patients had reactivity with similar sequences in the fibronectin-1^2015–2029^ and *Bb* p93^101–125^ epitopes (the results in the 2 patients with positive responses are shown in Figure 3B). PBMCs from the other 2 patients had the highest responses to collagen Vα1^1730–1750^ and BBQ62^71–85^ epitopes (their results are shown in Figure 3I). For these experiments, the patients who had reactivity with the fibronectin epitope were numbered 1 and 2, and the patients who had responses to the collagen epitope were numbered 3 and 4.

**Figure 4:**
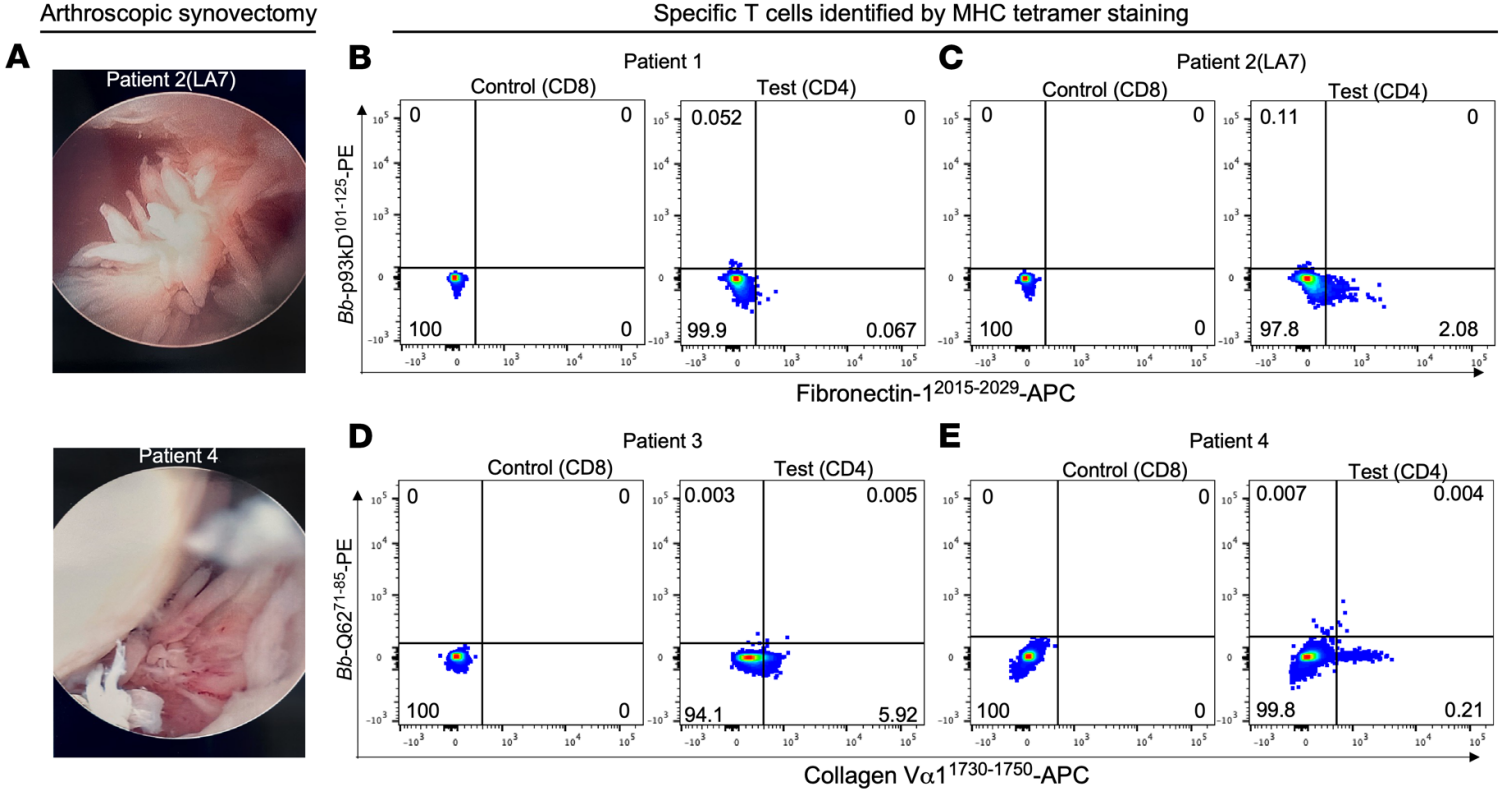
HLA-DR tetramer staining of ECM-specific or *Bb*-specific T cells in SFMCs. In 4 patients who had the HLA-DRB1*1501 allele and ECM-reactive and *Bb*-reactive T cells in ELISpot assays, SFMCs were analyzed using tetramer reagents. (**A**) The patients had massive synovial fibroblast proliferation, shown here at the time of synovectomy in patients 2 and 4. (**B**–**E**) SFMCs of 4 LA patients, 2 each who had ELISpot reactivity with the fibronectin-1^2015–2029^ and *Bb*-p93^101–125^ peptide pair (**B** and **C**) or with the collagen Vα1^1730–1750^ and BBQ62^71–85^ peptide pair (**D** and **E**), were stained with DRB1*1501 tetramers containing each of these peptides. In 3 of the 4 patients (nos. 2, 3, and 4), SFMCs showed tetramer staining for fibronectin-1–specific T cells (**C**) or for collagen Vα1–specific T cells (**D** and **E**). No staining was seen in any patient for *Bb*-derived peptides or for double-positive cells. CD8^+^ T cells, which do not bind HLA-DR molecules, were used as negative controls [Control (CD8)] to define cutoffs for HLA-DR binding of CD4^+^ T cells [Test (CD4)]. Flow cytometry data were determined by BD FACSAria Fusion.

SFMCs in these 4 patients were obtained from 2 to 6 months after the completion of antibiotic therapy; at that time, PCR testing for *Bb* DNA in SF was negative. Based on subsequent follow-up, the 4 patients were at the far end of the spectrum for severity and duration of postinfectious LA. Because of incomplete responses to methotrexate, 3 of the 4 patients (nos. 2, 3, and 4) elected to have arthroscopic synovectomies after 18–34 months of postinfectious LA. Examples of their massive synovial hypertrophy are shown in Figure 4A. However, only synovial tissue from patient 2 (who was LA7) was examined for HLA-DR–presented peptides by nano-LC-MS/MS (Supplemental Figure 1). The final patient (patient 1) was treated with methotrexate and then a TNF inhibitor, etanercept, and had resolution of arthritis 2 years after the completion of antibiotic therapy.

In preparation for tetramer studies, SFMCs were first stimulated with the fibronectin^2015–2029^ or collagen^1730–1750^ peptide or with each corresponding *Bb*-mimic peptide in culture for 14 days to expand any cell population that was present. DRB1*1501 tetramers containing each sequence were then used to examine binding with patients’ SFMCs. The gating strategy is given in Supplemental Figure 2. Not enough cells were still available from the appropriate patient to test the laminin B2 epitope and its *Bb*-mimic epitope by flow cytometry.

In patient 2, a small but discernible population of DRB1*1501-fibronectin^2015–2029^ CD4^+^ tetramer-binding cells was detected, which were not found among control CD8^+^ gated T cells (Figure 4C). This CD4^+^ T cell population was not seen in patient 1 (Figure 4B). These results were consistent with their ELISpot values; patient 1 had 18 spot-forming units (SFU)/10^6^ cells, whereas patient 2 had 32 SFU/10^6^ cells (Figure 3B). DRB1*1501–*Bb* p93^101–125^ tetramer-binding T cells were not seen in either patient 1 or 2 (Figure 4, B and C). In patients 3 and 4, DRB1*1501–collagen Vα1^1730–1750^ tetramer-binding CD4^+^ T cells were detected, though the tetramer staining was much stronger for patient 4 than for patient 3 (Figure 4, D and E). These results were also consistent with their ELISpot values; patient 3 had 22 SFU/10^6^ cells, whereas patient 4 had 130 SFU/10^6^ cells (Figure 3I). DRB1*1501-BBQ62^71–85^ tetramer-binding T cells were not seen in either patient 3 or 4. Thus, tetramer staining directly identified ECM-autoreactive CD4^+^ T cells in 3 of the 4 patients tested but did not detect T cells specific for *Bb*-mimic epitopes in any patient.

### Identification of subtypes of ECM-autoreactive CD4^+^ T cells.

In patients 2 and 4, enough SFMCs remained to determine the effector/regulatory subtypes of ECM-autoreactive CD4^+^ T cells. For this purpose, T cells specific for the fibronectin^2015–2029^ or collagen Vα1^1730–1750^ epitope were identified using HLA-DR tetramers, and each CD4^+^ T cell subtype was stained separately using antibodies to identify intracellular expression of T-bet, RoRγt, or FoxP3. The gating strategy was the same as that used above for identification of HLA-DR tetramer binding (Supplemental Figure 2).

**Figure 5:**
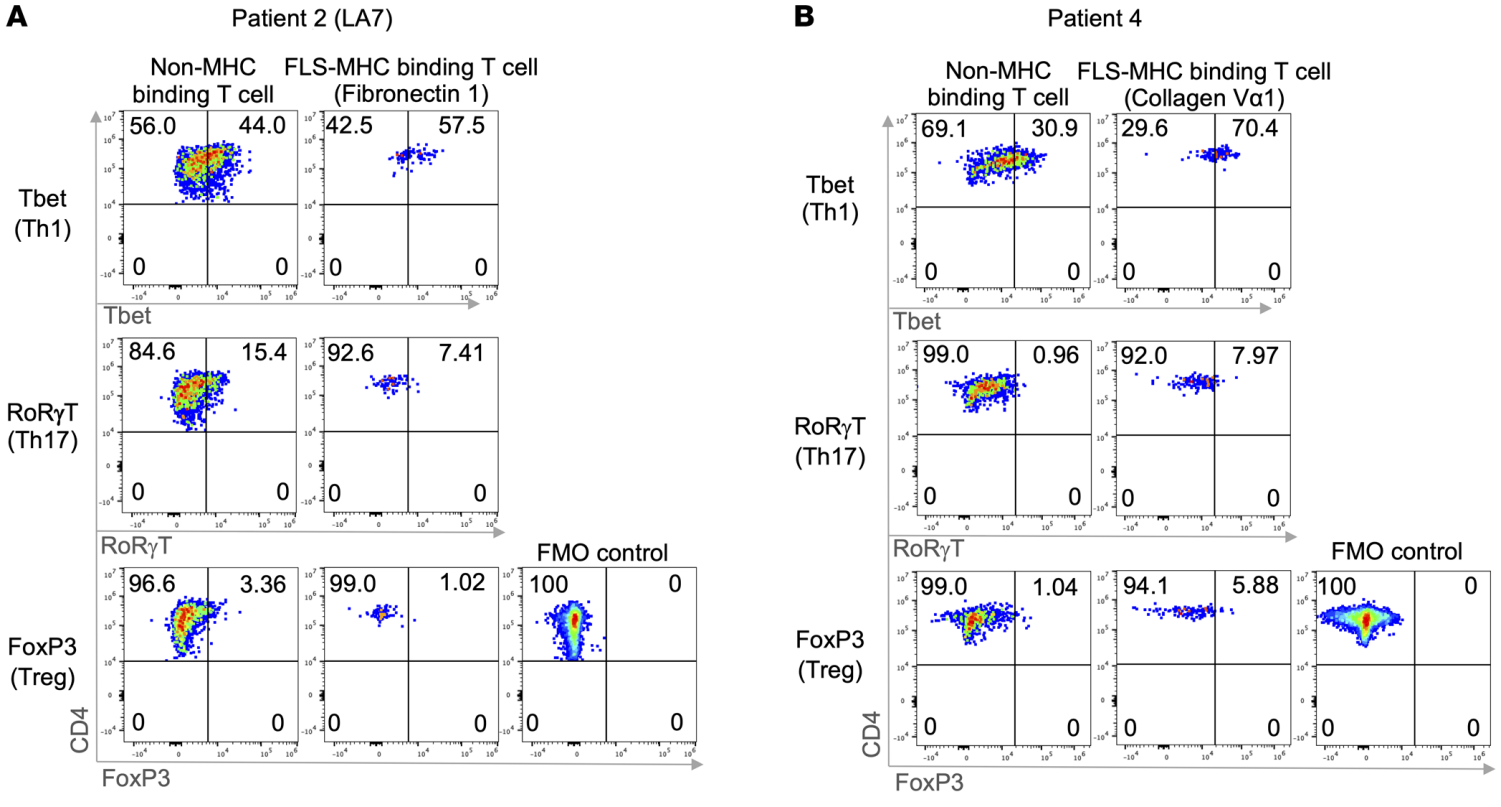
Intracellular staining for ECM autoantigen–specific CD4^+^ T cell subtype identification. In patient 2 (**A**) and patient 4 (**B**), intracellular staining of ECM-specific CD4^+^ T cell subtypes is shown using antibodies against T-bet, RoRγt, and FoxP3. Flow cytometry data were determined using the Cytek Aurora.

In both patients, each tetramer representing a single T cell epitope identified a mixed CD4^+^ T cell subtype population (Figure 5A). In patient 2, the highest percentages of cells reactive with the fibronectin^2015–2029^ epitope (57.5%) were T-bet–positive Th1 cells, a small percentage of cells were RoRγt-positive Th17 cells (7.41%), and a minimal percentage (1.02%) were FoxP3-expressing Tregs. Patient 4, whose cells reacted with the collagen Vα1^1730–1750^ epitope, had similar findings (Figure 5B). The highest percentages of collagen-reactive cells (70.4%) were T-bet–positive Th1 cells, 7.97% were RoRγt-positive Th17 cells, and 5.88% were FoxP3-expressing Tregs. These observations suggest that CD4^+^ T cells in both patients would likely play a role in enhancing and sustaining inflammatory Th1 and Th17 cell immune responses in joints, whereas antiinflammatory responses would seem minimal.

## Discussion

In this study, using immunopeptidomics to identify HLA-DR–presented peptides directly from postinfectious LA patients’ synovial tissue, we identified T cell epitopes of 3 ECM proteins, likely presented primarily by fibroblast-like synoviocytes (FLSs), a cell of central importance in the pathogenesis of autoimmune, chronic inflammatory forms of arthritis (7). Of patients with postinfectious LA, 58% had T cell responses and 17% had antibody responses to fibronectin-1, laminin B2, and/or collagen Vα1. T and B cell responses to the 3 synovial ECM proteins were found almost exclusively in patients with postinfectious LA, which is the period of massive fibroblast proliferation in synovial tissue. In contrast, T and B cell responses to the 3 previously identified vascular-associated autoantigens (ECGF, annexin A2, and apoB-100) may develop during spirochetal dissemination. Therefore, these responses may be found in antibiotic-responsive LA patients seen prior to antibiotic therapy as well as in postinfectious LA patients (15–17).

These vascular and ECM autoantigens are found at important sites of *Bb* infection. In archival synovial tissue from untreated LA patients, a few spirochetes were seen in and around damaged blood vessels and in the ECM aligned with collagen fibrils (23–25). To explain these tropisms for joints, *Bb* has multiple surface adhesins that attach to host integrins, glycoproteins, and glycosaminoglycans (26, 27). Of particular importance, *Bb* decorin-binding protein binds host decorin, a glycosaminoglycan that “decorates” collagen fibrils (28), and spirochetes colonize native type I collagen lattices directly (29), which are abundant in sites of *Bb* persistence (24, 30). Type V collagen, which is a minor component of type I collagen fibers, caps the ends of the fibers and is essential for fibrillary formation and tissue quality (31).

Four mechanisms have been proposed to explain the development of infection-induced autoimmunity: molecular mimicry between microbial and self-epitopes, bystander activation of autoreactive T cells, release of cryptic epitopes in damaged tissue, and/or epitope spreading involving both microbial and self-proteins (32, 33). To test the molecular mimicry hypothesis, we searched for sequence alignment between ECM and *Bb-*mimic peptides and found examples of possible interest. However, with tetramer reagents, the best example, which was between collagen Vα1 and BBQ62-like epitopes, showed binding only to the collagen Vα1 epitope and not to the BBQ62 epitope. Similarly, antibody responses could be demonstrated only to a recombinant collagen Vα1 protein and not to a recombinant BBQ62-like protein. In addition, the bystander activation hypothesis seems unlikely because of the strong HLA-DR association with T cell responses to ECM epitopes. The remaining possibility is the model of epitope spreading involving both *Bb* and self-proteins, perhaps including cryptic epitopes of damaged host tissues. We think that this model fits with current observations. The ECM of joints is a major site of spirochetal infection, there is a strong HLA-DR association with ECM autoimmune responses, and molecular mimicry between *Bb* and self-ECM epitopes does not appear to be involved.

Accordingly, we hypothesize that both *Bb*-derived adhesins and damaged ECM proteins at this prominent site of *Bb* infection are presented by professional and non-professional APCs within the highly inflammatory environment of the joint (34–37). In a subset of patients, primarily those with DRB1*04 or DRB1*1501 alleles, excessive immune responses to *Bb* antigens lead to a break in immune tolerance with epitope spreading from *Bb* antigens to infection-associated tissue antigens, such as fibronectin, laminin, and collagen. As shown here and in our previous studies (14), hundreds of self-peptides are presented by HLA-DR molecules in postinfectious LA synovial tissue, but only a few, such as the ECM peptides identified here, were immunogenic. ECM-reactive T cells presumably recognize their autoantigens at low affinity, and therefore underwent incomplete negative selection in the thymus. While immunologically inactive during homeostasis, the highly inflammatory milieu from the infection would promote chemoattraction to and activation of these T cells in affected joints.

We propose that these ECM-autoreactive T effector cells are drivers of persistent inflammatory synovitis, resulting in continued release of ECM autoantigens and a feedback loop of autoimmune inflammation. In addition, cytotoxic immune responses to vascular-associated autoantigens may lead to obliterative microvascular lesions in synovial tissue (19). These autoimmune inflammatory responses may be further enhanced by immune reactivity with spirochetal remnants, such as *Bb* peptidoglycan, which is uniquely difficult to clear (38). However, after the eradication of live spirochetes from joints with antibiotic treatment, autoimmune inflammation, with the help of disease-modifying antirheumatic drug (DMARD) therapies, eventually declines, and the arthritis usually resolves within 1 to 2 years (4).

RA synovial tissue also shows marked fibroblast proliferation, but as shown here, RA patients rarely had T cell reactivity with the T cell epitopes of ECM proteins identified here. In postinfectious LA, these responses are presumably shaped initially by *Bb* infection, whereas RA patients often have T cell responses to citrullinated collagen epitopes (39), perhaps shaped in some cases by mucosal immune responses to certain commensal organisms (40–42).

Limitations of our study include the necessarily small number of patients in whom HLA-DR–presented T cell epitopes were identified in synovial tissue and in whom tetramer reagents could be used to assess HLA-DR–peptide binding to autoreactive CD4^+^ T cells. However, with the epitopes identified, it was possible to test PBMCs from large patient and control groups for reactivity with peptides of interest using ELISpot assays. Second, we did not have the necessary early serial samples of PBMCs to explore further the epitope spreading hypothesis. In RA, epitope spreading to citrullinated proteins begins during the pre-arthritic, asymptomatic stage, and the process is nearly complete by the time clinically apparent disease develops (43). In our study, PBMCs from 1 postinfectious LA patient were collected 6 weeks before the start of i.v. antibiotic therapy, and this patient had T cell reactivity with epitopes of all 3 ECM proteins identified here, suggesting that such responses likely begin during the period of infection. Finally, although no mouse model duplicates all features of postinfectious LA, mouse models, such as the IL-10–knockout mouse (*Il-10^–/–^*) (44), may be valuable in further exploration of the infection-induced autoimmunity hypothesis proposed here.

In conclusion, the correlations shown here between ECM-autoreactive T cells and certain HLA-DR alleles, Th1 phenotype, and longer arthritis duration suggest that these autoimmune responses are an important component of disease pathogenesis. Along with the negative culture and PCR results from synovial tissue in these patients, the observations reported here support the treatment of such patients with DMARDs, including methotrexate and/or TNF inhibitors (4). Moreover, the identification of these T or B cell responses may provide biomarkers to help differentiate the infectious from the postinfectious periods of LA. Finally, this example of infection-induced autoimmunity may provide insights regarding pathogenetic factors in other forms of chronic inflammatory arthritis.

## Methods

### Patients and control subjects.

The 110 patients with Lyme disease met the criteria of the Centers for Disease Control and Prevention for *Bb* infection (45), and those with RA, spondyloarthropathies (SpA), or other connective tissue diseases, primarily lupus, met validated criteria for these diseases (46, 47). LA patients received antibiotic therapy according to an algorithm (3, 4), as detailed in the guidelines of the Infectious Diseases Society of America (48). PBMCs and SFMCs were cryopreserved in liquid nitrogen freezers (Taylor-Wharton K series). HLA typing of patients was performed in the Red Cross Laboratory in Dedham, Massachusetts, USA.

### Isolation and identification of HLA-DR–presented peptides.

We have previously published detailed methods for immunoprecipitation, elution, and identification of HLA-DR–presented peptides from synovial tissue (13, 14). Briefly, the tissue (8–10 g) was homogenized in lysis buffer, HLA-peptide complexes were obtained by immunoaffinity purification, HLA-DR–presented peptides were eluted in formic acid buffer, and peptide spectra were identified by nano-LC-MS/MS. Spectra-to-peptide assignments were made by searching of each patient’s MS/MS data set against a UniProt human database using 3 search engines: Mascot, Open Mass Spectrometry Search Algorithm (OMSSA), and X!Tandem. A consensus match among at least 2 programs was required for identification of a peptide sequence, and assignments were verified via manual inspection (by QW and CEC). The software pLabel was used to show the spectra of FLS-derived peptides (Supplemental Figure 1) (49).

### ELISpot T cell assay.

To determine T cell reactivity, HLA-DR–presented peptides identified from synovial tissue were synthesized (Mimitopes), and their immunogenicity was determined by stimulation with the matching patients’ PBMCs in IFN-γ enzyme-linked immunospot (ELISpot) assays (Cellular Technology Ltd.). Subsequently, the HLA-DR–presented peptides of ECM proteins were synthesized (GenScript) to determine their antigenicity with PBMCs from many patients using IFN-γ ELISpot assays. In addition to the epitopes of ECM proteins identified from patients’ synovia, the complete sequence of each of the 3 proteins was analyzed using the Immune Epitope Database (IEDB; http://tools.iedb.org/mhcii) to identify other predicted T cell epitopes of interest and their HLA-DR binding score (50). The peptide sequences identified from synovial tissue and selected predicted epitopes were synthesized and used to stimulate PBMCs from our cohort of LA patients and healthy control subjects.

Before stimulation, frozen PBMCs were thawed and rested overnight in RPMI 1640 medium supplemented with penicillin/streptomycin (Gibco). The next morning, cell viability was assessed by 0.4% trypan blue staining; the average viability was 94%. PBMCs (2 × 10^5^ cells per well) were first stimulated in duplicate using pools of 2 or 3 peptides (1 μM/well for each peptide), which were incubated in culture medium (Cellular Technology Ltd.) supplemented with glutamine (Gibco) at 37°C with 5% CO_2_ for 5 days. For analysis, the cells from each well were transferred to an ELISpot plate coated with IFN-γ antibodies and incubated overnight. Spots were counted using an ImmunoSpot Series 3B Analyzer (Cellular Technology Ltd.). Spot-forming units (SFU) more than 3 SD above the mean value in healthy control subjects were defined as a positive response. For positive controls, the mitogen PHA (Gibco) and PepMix antigen-specific T cell stimulator (JPT Peptide Technologies) were used in all experiments. For a negative control, unstimulated cells were included on each plate. For immunoreactive ECM-peptide pools, retesting, when enough cells were available, was done with single peptides contained in the pool.

### ELISA for serum IgG autoantibody determinations.

ELISA plates (96 wells) were coated with 5 ng/well of fibronectin-1 (Novoprotein), laminin B2 (Novoprotein), or collagen Vα1 (Abcam) overnight at 4°C. After washing with PBS containing 0.05% Tween-20 (PBST), the wells were incubated with blocking buffer (100 μL/well; Chondrex) for 1 hour. Afterward, serum samples (1:100, diluted in the blocking buffer) were added in duplicate wells and incubated for 1.5 hours, followed by HRP-conjugated goat anti-human IgG (1:2,000; AP112P, Sigma-Aldrich) and then tetramethylbenzidine substrate (BD). All incubations and washes were performed at room temperature. For internal control standardization, serum samples from 3 positive controls (high, intermediate, and low-positive) and 10 healthy control subjects were performed on each ELISA plate. OD values were determined at OD_450_ (iMarK microplate reader, Bio-Rad). OD values that were more than 3 SD above the mean values in healthy control subjects were considered positive.

### Molecular mimicry studies.

To identify possible T cell epitope mimicry between ECM and *Bb* peptides, we searched for sequence alignment between these microbial and self-peptides by BLAST analysis of *Bb* (taxid:139) (https://blast.ncbi.nlm.nih.gov/Blast.cgi). Candidate *Bb*-mimic peptides were then analyzed by the IEDB for HLA-DR binding prediction. The *Bb*-mimic peptides were synthesized (GenScript) and tested for reactivity in IFN-γ ELISpot assays using patients’ PBMCs, which had previously shown positive responses in initial testing of pooled ECM peptides.

### MHC II tetramer and flow cytometry analyses.

As detailed in the Kwok protocol (51), on day 1, 5 × 10^5^ to 10 × 10^5^ SFMCs per well were loaded into 96-well U-bottom plates containing 200 μL of culture medium with peptide (1 μg/mL). The cells were stimulated with each ECM peptide or corresponding *Bb*-mimic peptide and cultured in RPMI 1640 with l-glutamine (Sigma-Aldrich) supplemented with 10% human serum (Sigma-Aldrich), 1% penicillin/streptomycin (Gibco), 0.1% 2-mercaptoethanol (Gibco), and 20 U/mL of recombinant human IL-2 (R&D Systems). Anti-CD3/28 Dynabeads (Gibco) were used as a positive control to monitor T cell expansion. On days 3 and 6, culture medium was replaced by resuspending of cells in fresh culture medium. On day 7, cells were transferred into 48-well plates with fresh culture medium (total volume, 0.5 mL/well). At day 14, SFMCs were harvested and prepared for MHC tetramer staining.

HLA-DRB1*1501 tetramers containing the collagen or fibronectin peptide were labeled with allophycocyanin and the corresponding *Bb*-mimic peptides were labeled with phycoerythrin, according to a previously published protocol (52). Briefly, for MHC tetramer staining, 1 × 10^7^ SFMCs, which had been grown 14 days in culture, were resuspended in 200 μL of sorting buffer (SB: PBS with 2% FBS and 0.1% NaN_3_) in 15 mL centrifuge tubes. Two microliters of each tetramer and human Fc blocking reagent (BD Biosciences) were added to each tube and incubated at room temperature for 1 hour, followed by 1 wash in 15 mL of cold SB (4°C) at 400*g* for 5 minutes. For tetramer enrichment, anti-phycoerythrin or anti-allophycocyanin (Miltenyi Biotec) was added to SFMCs in cold SB (total volume 150 μL) and incubated at 4°C for 20 minutes. After washing again, 3 mL of each tube was loaded on a Miltenyi LS magnetic column for positive selection. For antibody staining, enriched tetramer-binding SFMCs were incubated for 30 minutes with fluorescent-conjugated antibodies against surface markers (Supplemental Table 2). After washing twice in 4 mL of cold SB, the stained SFMCs were resuspended in 1 mL of 4% paraformaldehyde (BD Cytofix/Cytoperm) solution at 4°C for 15 minutes followed by 2 washes with SB and resuspension in 1 mL BD Perm/Wash buffer (PW) for 15 minutes. For intracellular staining, the cells were washed again and resuspended in 100 μL of PW, and appropriate antibodies were added to individual tubes (Supplemental Table 2) followed by incubation at 4°C for 30 minutes. After 2 washes with 1 mL PW, the cells were resuspended in 300 μL of SB and analyzed by flow cytometry (BD FACSAria Fusion for cell frequencies or Cytek Aurora for intracellular staining analysis).

### Statistics.

Quantitative data were analyzed using an unpaired, 2-tailed *t* test with Welch’s correction or Mann-Whitney test, which are tests for non-parametric data; and paired samples were analyzed using Wilcoxon’s rank-sum test. Categorical data were analyzed by Fisher’s exact test, and correlations were determined using Spearman’s correlation test. All analyses were performed using GraphPad Prism 8 (GraphPad Software). All *P* values are 2-tailed, and *P* values less than or equal to 0.05 were considered statistically significant.

### Study approval.

The study was approved by the Human Investigations Committee at Massachusetts General Brigham Hospitals. All patients and parents of teenagers aged 12–18 and control subjects provided written informed consent prior to their participation in the study.

### Data availability.

The tandem mass spectra of the ECM proteins studied here (Table 1) are shown in Supplemental Figure 1. Labeled tandem mass spectra for all peptides identified by at least 2 protein databases as well as peptides identified by 1 protein database with the assignment approved upon manual inspection have been published previously (14) and are available on the American Chemical Society publications website at doi:10.1021/acs.jproteome.6b00386. The values of data points shown in Figures 1–3 are provided in the Supporting Data Values file. The frequencies of HLA-DR tetramer–specific, ECM-reactive CD4^+^ T cells for each of 4 individual patients, as determined by flow cytometry, are shown in Figure 4; and the frequencies of T-bet–, RoRγt-, and FoxP3-positive of ECM-binding CD4^+^ T cells for 2 individual patients, as determined by flow cytometry and intracellular staining, are shown in Figure 5. The gating strategies for determinations of these cell populations are shown in Supplemental Figure 2.

## Author contributions

KK helped design the study, performed experiments, and analyzed data. KS helped design the study and obtained and processed synovial tissue. RBL helped design the study, performed experiments, and analyzed data. LMM and AP performed initial ELISpot assays to identify immunogenic HLA-DR–presented peptides in synovial tissue. SLA and ACS enrolled and cared for patients. QW and CEC did the mass spectrometry analyses. DEK and CAD performed ELISAs for certain *Bb* or host proteins. LC and DEK helped with data analysis and production of figures. AGB examined the sequences and potential expression of *Bb* protein BBQ62, which was encoded on *Bb* lp56, and also found a BBQ62-like protein encoded on lp28-2. JJM designed the flow cytometry experiments and prepared the tetramer reagents. ACS helped design the study and provided advice and data interpretation. All authors participated in the writing of the manuscript and approved the final version of this article.

## Supplementary Material

Supplemental data

ICMJE disclosure forms

Supporting data values

## Figures and Tables

**Table 4:**
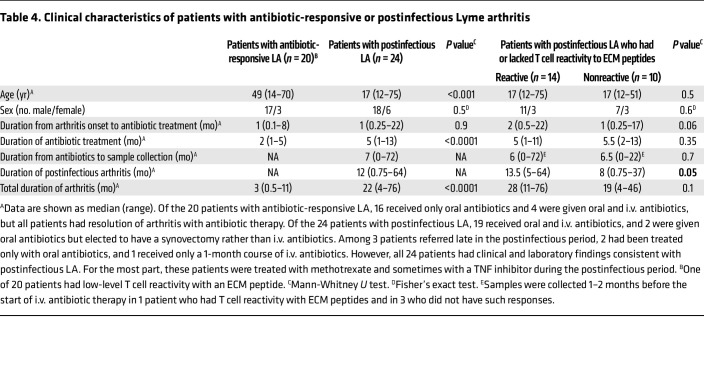
Clinical characteristics of patients with antibiotic-responsive or postinfectious Lyme arthritis

**Table 5:**
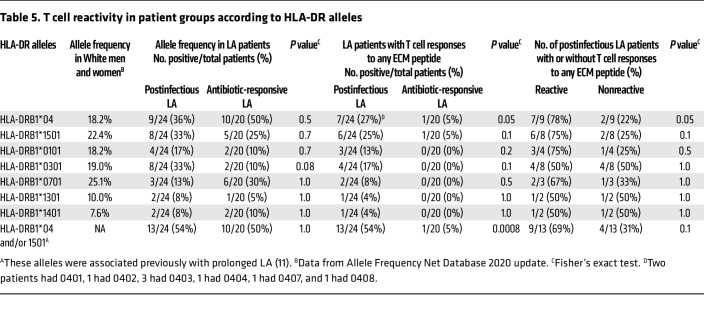
T cell reactivity in patient groups according to HLA-DR alleles

**Table 3:**
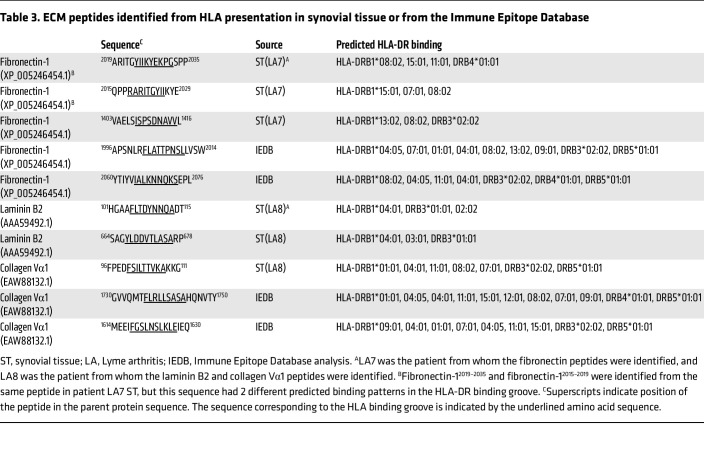
ECM peptides identified from HLA presentation in synovial tissue or from the Immune Epitope Database

**Table 2:**
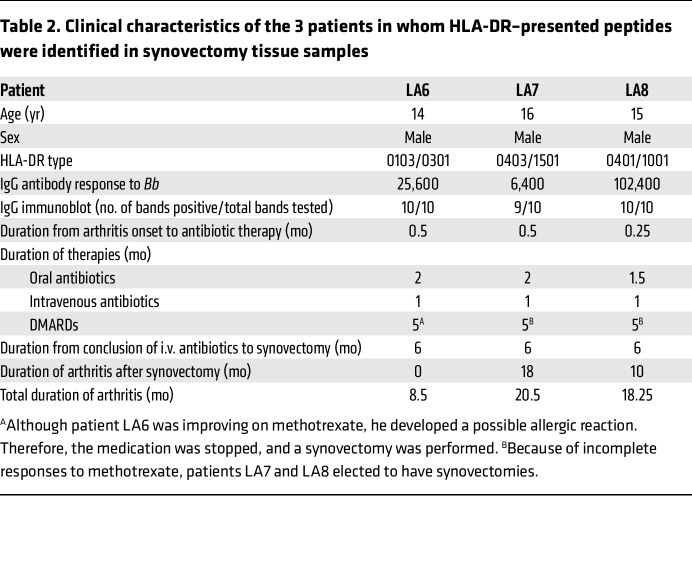
Clinical characteristics of the 3 patients in whom HLA-DR–presented peptides were identified in synovectomy tissue samples

**Table 1:**
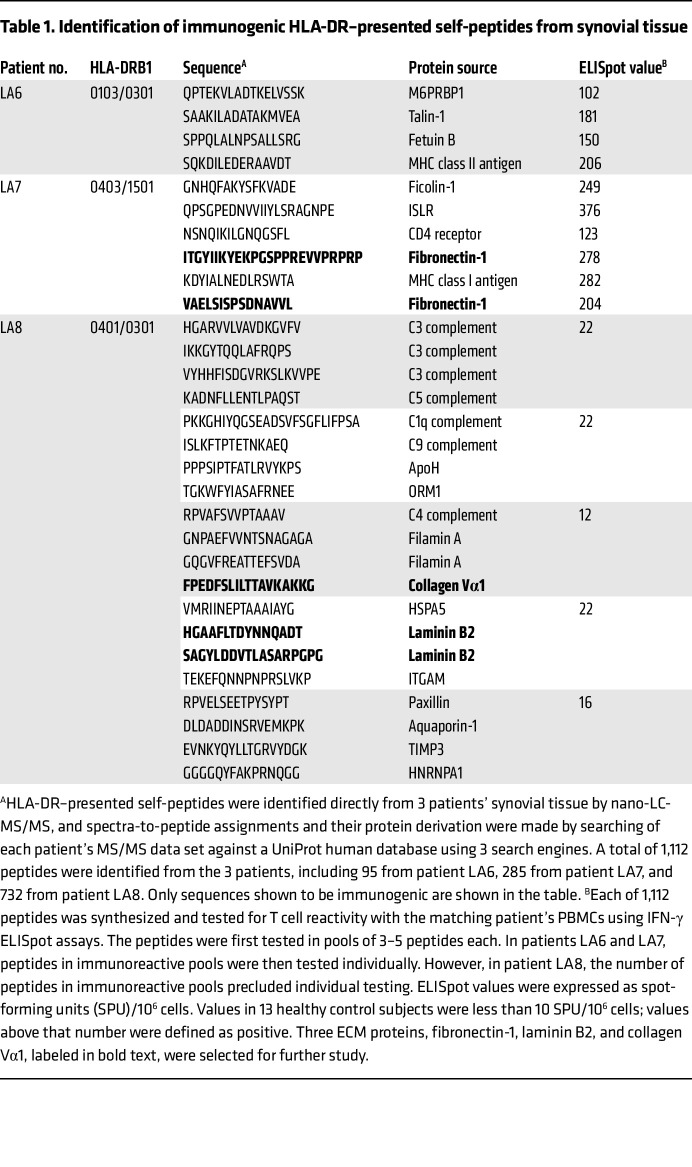
Identification of immunogenic HLA-DR–presented self-peptides from synovial tissue

## Version 1 07/20/2023

In-Press Preview

## Version 2 09/01/2023

Electronic publication
