# Supplementary material for: Autoimmunity to synovial extracellular matrix proteins in patients with postinfectious Lyme arthritis
Source: J Clin Invest. 2023 Sep 1;133(17):e161170. doi: 10.1172/JCI161170 (PMC10471169; doi:10.1172/JCI161170)
Supplement: Supplemental data [file jci-133-161170-s089.pdf]

Supplemental data

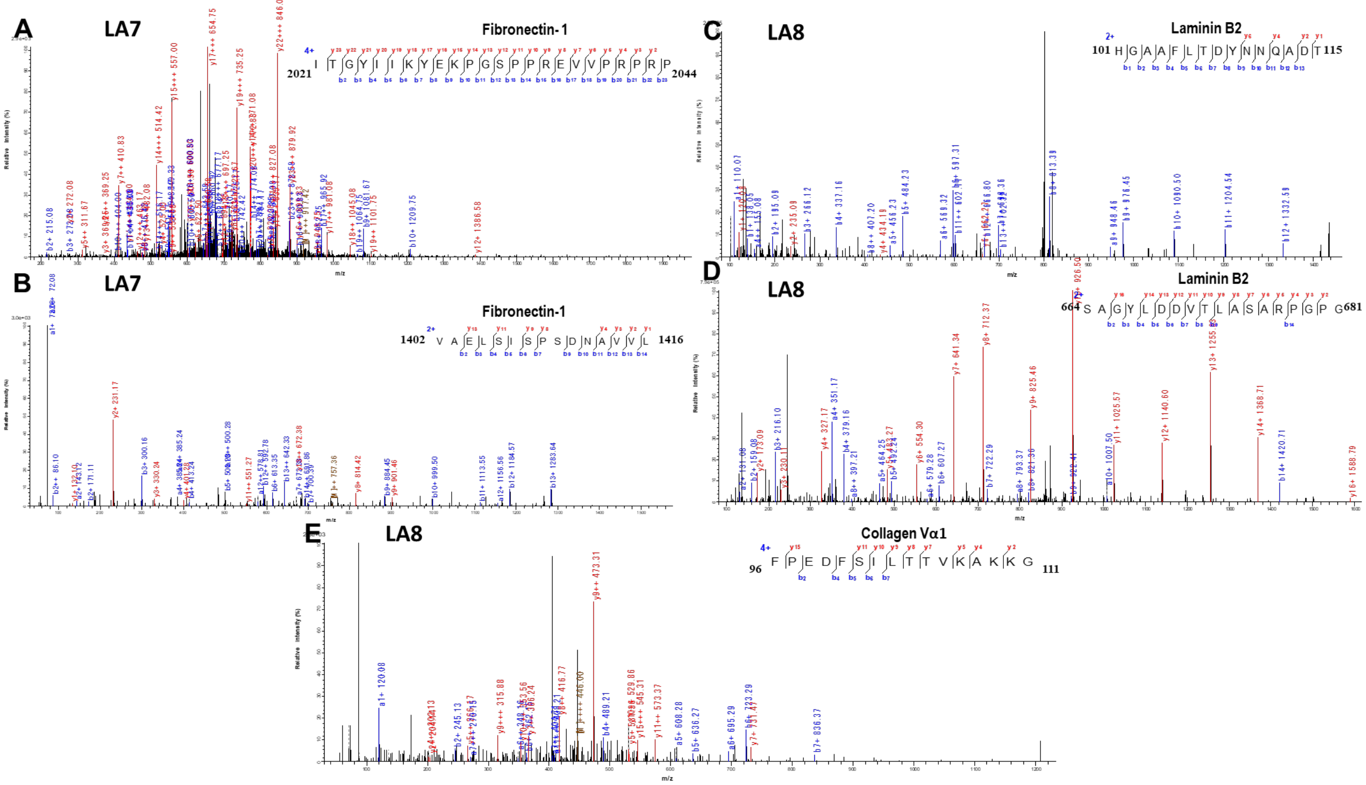

Supplemental Figure 1. Tandem mass spectra for HLA-DR-presented, ECM peptides identified in synovial tissue of 2 LA post-infectious LA patients (LA7 and LA8).

The peptide ITGYIIKYEKPGSPPREVVPRPRP was consistently identified from LA7 by OMSSA and X!Tandem from CID MS2 spectrum recorded using an LTQ-Orbitrap XL MS (**A**). VAELSISPSDNAVVL was consistently identified from LA7 by Mascot, OMSSA and X!Tandem from CID MS2 spectrum recorded using a 6550 QTOF MS (**B**). HGAAFLTDYNNQADT was consistently identified from LA8 by Mascot, OMSSA, and X!Tandem from HCD MS2 spectrum recorded with a Q Exactive plus MS (**C**). SAGYLDDVTLASARPGPG was consistently identified from LA8 by Mascot, OMSSA, and X!Tandem from HCD MS2 spectrum recorded with a Q Exactive plus MS (**D**). FPEDFSILTTVKAKKG was consistently identified from LA8 by OMSSA and X!Tandem from CID MS2 spectrum recorded using a 6550 QTOF MS (**E**). The fragment ion error tolerance was 0.5 Da for data acquired on the LTQ-Orbitrap XL MS, 0.05 Da for data acquired on the 6550 QTOF MS, and 0.02 Da for data acquired on the Q Exactive plus MS.

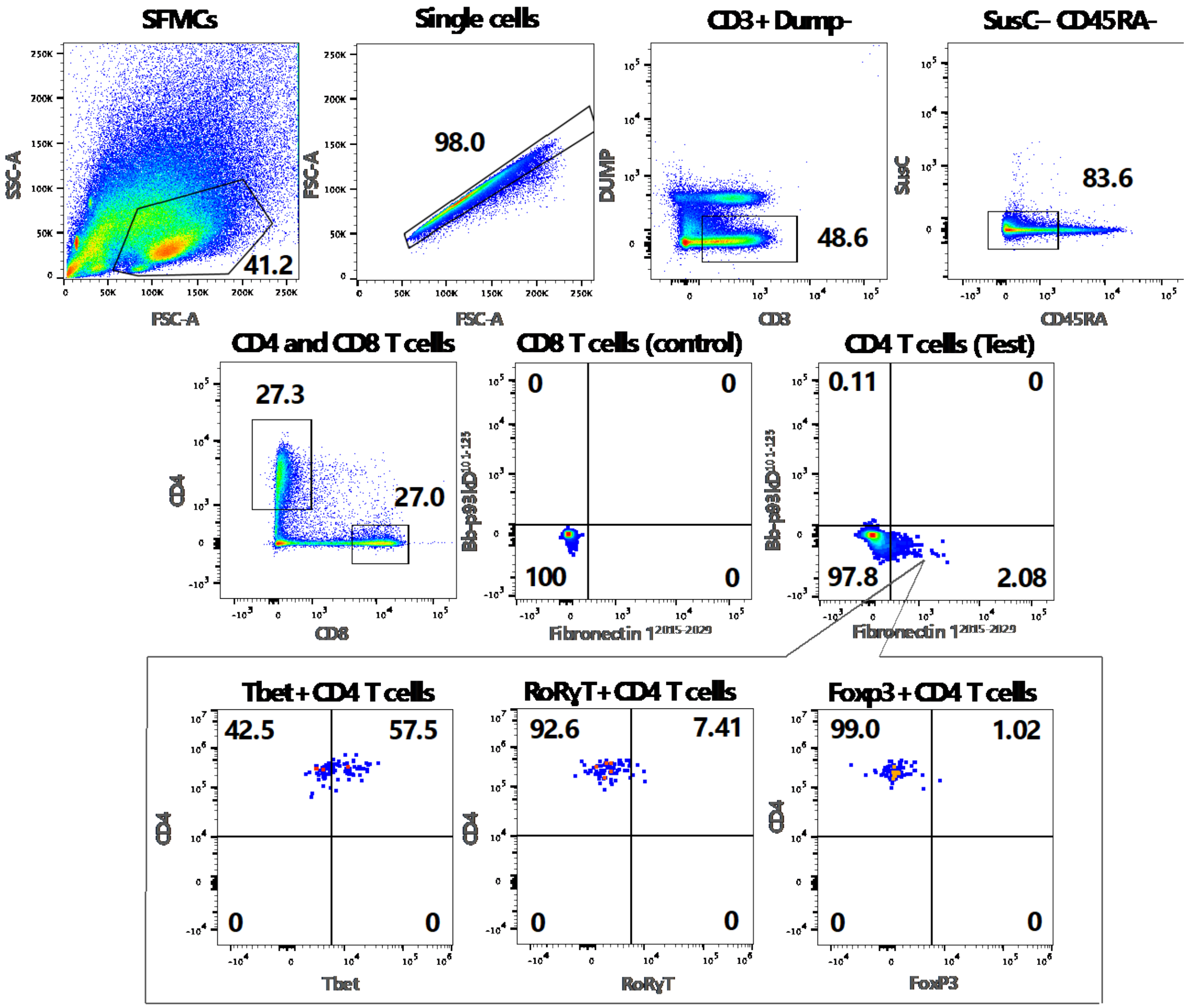

Supplemental Figure 2. Gating strategy for T cell subset identification.

The gating was started by SFMC followed by singlet cell using FSC and SSC; CD14, CD20 (Dump) negative CD3 positive T cells were gated, and the SusC negative-CD45RA- T cells were selected. Next, CD4 and CD8 T cells were gated for HLA-DR tetramers containing either the FLS or *Bb*-mimic peptide. For further intracellular protein analysis, T-bet, RoRyt and Foxp3 were analyzed in the FLS-specific T cell population.

Supplemental Table 1. ECM peptides and putative Bb-mimic peptides

Pair No.	Peptide name (NCBI accession no.)	Sequence ±	Host-Bb peptide mimicry	HLA-DR alleles predicted to bind both host and *Bb* peptides	HLA alleles in patients who had responses to both the host and *Bb*-peptides.
**1**	Fibronectin $^{2019-2035}$<br>(XP_005246454.1)<br>Bb-93kD $^{101-125}$<br>(CAA49829.1)	^{2019}ARITGYIIKYEKPGSPP^{2035}<br>^{101}LDSILNLRRILTGYIIKSFD^{125}<br>p-2,-1,1,2,3,4	Unlikely	DRB1*15:01, 08:02, 07:01, 13:02, DRB4*01:01	*Patient A: DRB1*15:01, 04:03
**2**	Fibronectin $^{2015-2019}$<br>(XP_005246454.1)<br>*Bb*-93kD $^{101-125}$<br>(CAA49829.1)	^{2015}QPPRARITGYIIKYE^{2029}<br>^{101}LDSILNLRRILTGYIIKSFD^{125}<br>p1, 5,6,7,8,9,10	**Possible**	DRB1*15:01, 08:02, 07:01, 13:02,	*Patient A: DRB1*15:01, 04:03<br>Patient B: DRB1*15:01, 07:01
**3**	Fibronectin $^{1403-1416}$<br>(XP_005246454.1)<br>*Bb* uncharacterized protein [CA8] $^{9-23}$<br>(EOA80158.1)	^{1403}VAELSISPSDNAVVL^{1416}<br>^{9}IAILLISPSCSTNNN^{23}<br>p-4, -2, 1,2,3,4	Unlikely	DRB1*08:02	None
**4**	Fibronectin $^{1996-2014}$<br>(XP_005246454.1)<br>*Bb*-transcriptional activator, putative, Baf family [JD1] $^{163-177}$<br>(>ADQ30717.1)	^{1996}APSNLRFLATTPNSLLVSW^{2014}<br>^{163}IKKFP I STPNSLLER^{177}<br>p1, 5,6,7,8,9,10	**Possible**	DRB1*07:01, *13:02	Patient B: DRB1*15:01, 07:01<br>Patient C: DRB1*15:01, 04:07
**5**	Fibronectin $^{2060-2076}$<br>(XP_005246454.1)<br>*Bb*-exodeoxyribonuclease V subunit beta$^{334-351}$<br>(>WP_002665669.1)	^{2060}YTIYVIALKNNQKSEPL^{2076}<br>^{334}TIDQNYIISNLKNYLKSE^{351}<br>p3,4,5, 8,9,10	Unlikely	DRB1*08:02, 11:01	None
**6**	Laminin B2 $^{101-115}$<br>(>AAA59492.1)<br>*Bb*-93kD $^{694-708}$<br>(CAA49829.1)	^{101}HGAAFLTDYNNQADT^{115}<br>^{694}KNLVILDVNTLKKVK^{708}<br>p4, 6	Unlikely	DRB1*03:01, *04:01, DRB3*01:01	Patient D: DRB1*04:08, 15:01
**7**	Laminin B2 $^{664-678}$<br>(>AAA59492.1)<br>*Bb*- DUF685 domain-containing protein$^{180-194}$<br>(WP_012663408)	^{664}SAGYLDDVTLASARP^{678}<br>^{180}NMEYNDDVTLIFSKS^{194}<br>p1, 3,4,5,6,7	**Possible**	DRB1*03:01, *04:01, DRB3*01:01	Patient B: DRB1*15:01, 07:01<br>Patient E: DRB1*01:01, 14:01<br>Patient F: HLA unknown
**8**	Collagen Vα1 $^{96-111}$<br>(EAW88132.1)<br>Bb-hypothetical protein L144_03550 [CA382] $^{116-130}$<br>(WP_031558192.1)	^{96}FPEDFSILTTVKAKKG^{111}<br>^{116}DLDFSILSSDSLKAK^{130}<br>p-1,1,2,3,4, 11	Unlikely	DRB1*04:01, *01:01, 07:01,	None
**9**	Collagen Vα1 $^{1730-1750}$<br>(EAW88132.1)<br>Bb-Q62$^{71-85}$<br>(>PNL87345.1)	^{1730}GVVQMTFLRLLSASAHQNVTY^{1750}<br>^{71}QAFVKLLSASASRFS^{85}<br>p1, 4,5,6,7,8,9	**Likely**	DRB1*01:01, *04:01, *04:05, *08:02, *09:01, *12:01, *15:01, *07:01, *11:01, DRB5*01:01, DRB4*01:01	Patient B: DRB1*15:01, 07:01<br>Patient C: DRB1*15:01, 04:07<br>Patient D: DRB1*04:08, 15:01<br>Patient G: DRB1*13:02, 13:03<br>Patient H: DRB1*03:01, 10:01
**10**	Collagen Vα1 $^{1614-1630}$<br>(EAW88132.1)<br>Bb- transcription elongation factor GreA$^{79-93}$<br>(>WP_038377009.1)	^{1614}MEEIFGSLNSLKLEIEQ^{1630}<br>^{79}QQFLTKRLNSLMLEI^{93}<br>p4,5,6,7, 9,10,11	Unlikely	DRB1*07:01, *15:01	*Patient A: DRB1*15:01, 04:03

*Patient A is LA7, the initial patient from whom the HLA-DR presented fibronectin peptides were identified in his synovial tissue.

±The location of the peptide in the protein is shown with superscript numbers. Matching same amino acids in the FLS-derived and *Bb*-mimic peptides are shown with a grey background. The predicted amino acids in the p1 through p9 locations in the HLA-DR binding groove are shown below the amino acid sequences.

Supplementary Table 2. Antibodies for flow cytometry

Protein/CD marker	Fluorescent	Clone
Surface marker		
CD45RA (Biolegend)	FITC	HI100
CD3 (Biolegend)	APC-Alex700	OKT3
CD4 (Biolegend)	APC-Cy7	RPA-T4
CD8 (Biolegend)	BV510	RPA-T8
CD14 and CD20 (Biolegend) for dump staining	PerCP-Cy5.5	M5E2 and 2H7
SusC (Moon's laboratory) for MHC negative gating	PE-Cy7	N/A
MHC 1501 tetramer with fibronectin$^{2015-2019}$ or collagen VαI$^{1730-1750}$ peptide	APC	N/A
MHC 1501 tetramer with *Bb*-mimic peptide of the fibronectin or collagen peptide	PE	N/A
Fc blocking reagent (BD)	-	564220[#]
Intracellular marker		
Foxp3 (Biolegend)	PE/Dazzle™ 594	206D
Tbet (Biolegend)	PE/Dazzle™ 594	4B10
RoRγt (BD)	PE-CF594	Q31-378

Catalogue number.
